# Sustained heterologous gene expression in pancreatic islet organoids using adeno-associated virus serotype 8

**DOI:** 10.3389/fbioe.2023.1147244

**Published:** 2023-07-19

**Authors:** Anna Voznesenskaya, Per-Olof Berggren, Erwin Ilegems

**Affiliations:** The Rolf Luft Research Center for Diabetes and Endocrinology, Karolinska Institutet, Stockholm, Sweden

**Keywords:** pseudoislet, AAV, adenovirus, gene therapy, delta cells, diabetes

## Abstract

Genetic modification of pancreatic islet organoids, assembled *in vitro* prior to transplantation is an emerging alternative to direct *in vivo* genetic manipulations for a number of clinical and research applications. We have previously shown that dispersion of islet cells followed by re-aggregation into islet organoids, or pseudoislets, allows for efficient transduction with viral vectors, while maintaining physiological functions of native islets. Among viruses currently used for genetic manipulations, adeno-associated viruses (AAVs) have the most attractive safety profile making them suitable for gene therapy applications. Studies reporting on pseudoislet transduction with AAVs are, however, lacking. Here, we have characterized in detail the performance of AAV serotype 8 in transduction of islet cells during pseudoislet formation in comparison with human adenovirus type 5 (AdV5). We have assessed such parameters as transduction efficiency, expression kinetics, and endocrine cell tropism of AAV8 alone or in combination with AdV5. Data provided within our study may serve as a reference point for future functional studies using AAVs for gene transfer to islet cell organoids and will facilitate further development of engineered pseudoislets of superior quality suitable for clinical transplantation.

## Introduction

Donor islet cell transplantation is an established therapy for insulin-dependent diabetes that is currently offered to patients who suffer from repeated episodes of hypoglycemia (Robertson, 2010; de Kort et al., 2011). Islet transplant recipients have improved glucose control, can stay free from exogenous insulin for up to several years post-transplantation and report increased quality of life. The main reasons that the procedure is reserved only for a restricted group of patients are the limited availability of good quality transplantation material, partial loss of islet mass following transplantation and challenges related to the host immune response. Patients receiving islet transplants need life-long immunosuppressive therapy while immunorejection and slow re-vascularization of the graft lead to islet cell death (Aghazadeh and Nostro, 2017). Overcoming these problems would allow to achieve sustained exogenous insulin independence, decrease the amount of donor material needed for successful transplantation and reduce the need for immunosuppressive therapy, and thus render islet transplantation accessible to more patients. Genetic modification of transplantation material could improve islet quality and supply islet cells with factors that provide protection from the immune attack or promote faster engraftment such as, for example, insulin-like growth factor 1 or vascular endothelial growth factor A (Shimoda et al., 2010; Mallol et al., 2017). Recently, the use of pseudoislets has shown a promise for highly efficient modification of islet cells (Friedlander et al., 2021).

Pseudoislets are islet cell organoids that can be prepared by dispersing and then re-aggregating islet cells following various experimental procedures. Large-scale production using orbital shakers assembles cells into pseudoislets of heterogenous sizes, while the use of either low-attachment multiwell plates or hanging drops allows to produce pseudoislets of homogenous size, although at a smaller scale (Friedlander et al., 2021). Pseudoislets retain endocrine cellular composition and major physiological functions of native islets both *in vitro* and after transplantation *in vivo* (van Krieken et al., 2019; Walker et al., 2020). Dispersion and re-aggregation allow creation of pseudoislets of controlled size and similar cellular composition. The procedure also allows to generate organoids of a smaller size that are generally better oxygenized and receive better nutrient supply throughout, both in culture and in the course of revascularization in case of transplantation (Lehmann et al., 2007; Zuellig et al., 2017).

Efficient gene transfer to islet cells is most easily achieved by the use of viral vectors. Adenoviruses (AdV), lentiviruses and adeno-associated viruses (AAV) have been used throughout the years to both study the role of various proteins in islet cell biology and in efforts to modify islet function for better transplantation outcomes (Lai et al., 2008). Viral transduction of islet cells reaches the highest efficiency if dispersed cells, as opposed to intact islets, are transduced (Friedlander et al., 2021). While transduction of intact pancreatic islets limits viral access to the outer cell layers of the islet, dispersion of islet cells enables virus particles to access all cells present within the islet. The procedure for pseudoislet generation therefore facilitates gene transfer into islet cells as single cells remain in suspension for hours during re-aggregation. Our previous work as well as the work of others show that, indeed, adenoviral vector-mediated transduction of dispersed islet cell suspension either before or during pseudoislet formation allows to achieve superior transduction efficiency (van Krieken et al., 2019; Walker et al., 2020).

Adenoviral vectors have been the most popular and well-studied tools in islet biology research involving gene transfer to pancreatic islet cells for more than 2 decades (Lai et al., 2008). However, expression of adenovirus-introduced genes is known to be relatively short-lived (van Krieken et al., 2019) and the vector itself induces cytotoxicity and inflammation at higher titers (Weber et al., 1997; Barbu et al., 2002; Barbu et al., 2005). These properties make adenoviruses unsuitable for gene therapy or *in vivo* studies when a long-term effect is desired.

In contrast, AAV vectors are non-pathogenic, have low immunogenicity, and their use results in long-term expression of the transferred gene in targeted cells (Hastie and Samulski, 2015). These vectors have also been successfully used in animal studies to transduce pancreatic islet cells *in vivo* (Wang et al., 2004; Wang et al., 2006; Jimenez et al., 2011). However, due to broad tissue tropism, cells within other tissues are also transduced when AAVs are introduced *in vivo* (Wang et al., 2006). As an alternative, pancreatic islets or pseudoislets can be transduced *ex vivo* prior to transplantation to avoid off-target transduction. Since most of the research has so far focused on *in vivo* use of AAVs, data on *in vitro* transduction of pancreatic islets with AAV vectors is relatively limited. In particular, AAVs have not been used in the past to transduce islet cell suspensions during pseudoislet formation.

In the current study, we have characterized in detail AAV serotype 8 (AAV8) performance in transduction of dispersed islet cells during pseudoislet formation. Our choice of AAV serotype was based on previous reports that demonstrated particularly high efficiency of AAV8 in transduction of endocrine islet cells (Wang et al., 2006; Cheng et al., 2007; Jimenez et al., 2011). We assessed transduction efficiency, cell tropism and kinetics of AAV8-driven expression in islet organoids and compared the majority of these parameters to those of human adenovirus 5 (AdV5), a viral vector commonly used to transduce islet cells.

## Materials and methods

### Animals

C57BL/6J mice were purchased from Charles River (Germany). All experiments were performed following Karolinska Institutet’s guidelines for care and use of animals in research and were approved by the animal ethics committee at Karolinska Institutet.

### Pancreatic islet isolation and generation of pseudoislets

Mouse pancreatic islets were isolated from C57BL/6J male mice by enzymatic digestion as described elsewhere (Li et al., 2009), and maintained in RPMI 1640 culture medium (Gibco) supplemented with L-glutamine (2 mM, GlutaMAX, Gibco), penicillin (100 U/mL, PenStrep, Gibco), streptomycin (100 μg/mL, PenStrep, Gibco) and 10% fetal bovine serum (Gibco). Human islets were obtained from the Nordic Network for Islet Transplantation and maintained in CMRL 1066 culture medium (Gibco) supplemented with HEPES (10 mM, Gibco), L-glutamine (2 mM, GlutaMAX, Gibco), sodium pyruvate (5 mM, Gibco), penicillin (100 U/mL), streptomycin (100 μg/mL, PenStrep, Gibco), nicotinamide (10 mM, Sigma-Aldrich) and 20% fetal bovine serum (Gibco). Islets were kept at 37 °C in a humidified atmosphere with 5% CO_2_ for a minimum of 24 h after isolation. For the generation of pseudoislets, islets were dissociated into single cells by enzymatic digestion for 10 min at 37°C using Accutase (Sigma-Aldrich). Cells were counted using a TC20 automated cell counter (Bio-Rad) and resuspended in serum-supplemented culture medium at a density of 1.25 × 10^4^ cells/mL. Cell suspension was distributed in CellCarrier spheroid ULA 96-well microplates (Perkin Elmer) at 200 µL/well. Pseudoislets were left to form in the CellCarrier plates for a minimum of 7 days.

### AAVs, adenoviruses and transduction

AAV8.CMV.TurboRFP preparation was purchased from Addgene (pENN.AAV.CMVs.TurboRFP.WPRE.RBG) and AdV5.CMV.EGFP was from Vector Biolabs (Cat. No: 1060). pENN.AAV.CMVs.TurboRFP.WPRE.RBG was a gift from James M. Wilson (Addgene viral prep # 105548-AAV8 and Addgene plasmid # 105548; http://n2t.net/addgene:105548; RRID:Addgene_105548).

The adenovirus AdV5.CMV.TurboRFP was prepared using the Gateway cloning system (Thermo Fisher Scientific) as follows. The product from PCR amplification of the plasmid encoding AAV8.CMV.TurboRFP, using the oligonucleotides 5′- CAC CAT GGT GAT GCG GTT TTG GCA GTA C -3′ and 5′- TCA CCC CGT CCT CGA GAC GGT ATC GAT GCG GGG -3′ as primers, was subcloned into pENTR/D. The resulting vector was sequenced and subjected to recombination with the promoter-less destination vector pAd/PL-DEST, for adenovirus production using HEK293A cells. The adenovirus AdV5.CMV.TurboRFP was then purified and concentrated using Fast-Trap (Millipore), following the manufacturer’s instructions.

For the transduction of pseudoislets, islet cell suspension was incubated with viral vectors during the first 72 h of pseudoislet formation. Final concentrations of the viruses in the cell suspension were as follows unless specified otherwise: 2.5 × 10^10^ vg/mL of AAV8.CMV.TurboRFP, 8.5 × 10^4^ PFU/mL of AdV5.CMV.TurboRFP and 8.5 × 10^4^–8.5 × 10^5^ PFU/mL of AdV5.CMV.EGFP.

The viral dose for AAV8.CMV.TurboRFP was chosen to be such as to induce a level of expression of TurboRFP that would be high enough to allow collection of the fluorescence signal from the core region of the pseudoislet 2 weeks post-transduction while using non-damaging excitation laser intensities. The time point of 10–14 days post-transduction used in most experiments was chosen for two reasons: first, to allow adequate comparison of AAV8.CMV.TurboRFP to AdV5.CMV.TurboRFP based on kinetics of TurboRFP fluorescence (Figures 2A, B), and second to avoid extended *in vitro* culture of pseudo(islets).

The amounts of AdV5.CMV.EGFP that we used to amplify AAV8-driven expression correspond to 6.8–68 MOI. We chose this concentration range based on the fact that in other cell models, MOIs of 1–50 provided enhancement of AAV transduction proportional to the amounts of AdV, with MOIs 50 to 100 providing maximal enhancement of AAV transduction (Fisher et al., 1996).

### Gene expression analysis

RNA was extracted from 25–30 pseudoislets using RNeasy Micro Kit (QIAGEN) following the manufacturer’s instructions. Reverse transcription was performed using Superscript II (Thermo Fisher Scientific) and the obtained full-length cDNA was amplified and purified as described previously (Picelli et al., 2014). Concentration of all samples were normalized prior to quantitative RT-PCR, which was performed using SYBR Green (Thermo Fisher Scientific) and a QuantStudio 5 system (Thermo Fisher Scientific). Primer sequences were 5′- CTA CCA GCT TCA TGT ACG -3′ and 5′- TCT TGA CGT TGT AGA TGA TG -3′ for *TurboRFP*, 5′- GAA CCG CAT CGA GCT GAA GG -3′ and 5′- CGT TGT GGC TGT TGT AGT TGT AC -3′ for *EGFP*, and 5′- TGC TGT TGG TGA TTG TTG GT -3′ and 5′- CTG GCT TGT GTG GGA AAG AT -3′ for *Tbp*. Expression of *TurboRFP* and *EGFP* are presented relative to *Tbp*.

### Laser scanning confocal microscopy

Laser scanning confocal micrographs were recorded at 12 bit using a Leica SP5 system equipped with ×25 water-immersion objective or at 16 bit with Leica SP8 system equipped with 10x air or ×40 water-immersion objectives (Leica Microsystems, Wetzlar, Germany). All confocal images are representatives and presented as maximum intensity projections in the XY plane or single confocal planes as indicated in figure legends.

### Immunohistochemistry (IHC)

For whole mount stainings, AdV5- and AAV8-transduced pseudoislets were collected 10–14 days post-transduction, briefly washed in PBS and fixed in 4% PFA for 1 h on ice, then washed in PBS followed by blocking overnight in PBS supplemented with 10% FBS and 0.3% Triton X-100. Pseudoislets were stained for 48 h with primary antibodies dissolved in blocking buffer: guinea pig anti-insulin (Cat. No. 16049, Progen, 1:1000), mouse anti-glucagon (G2654, Sigma, 1:500), rat anti-somatostatin (8330-0009, Bio-Rad, 1:500); and overnight with secondary antibodies dissolved in blocking buffer: goat anti-guinea pig Alexa 488 (Thermo Fisher Scientific, 1:1000), goat anti-mouse Alexa 488 (Thermo Fisher Scientific, 1:1000), goat anti-rat Alexa 488 (Thermo Fisher Scientific, 1:1000).

Cell death in forming pseudoislets was assessed by SYTOX green staining (Thermo Fisher Scientific). Pseudoislets were stained with 30 nM SYTOX green for 30 min prior to imaging. Whole mounts and SYTOX stained pseudoislets were imaged by confocal microscopy using Leica SP5 and SP8 systems, respectively, as specified above.

### Flow cytometry

Dissociated islet cells were first stained with fixable viability dye eFluor 780 (Thermo Fisher) at 1:1000 dilution in PBS for 30 min. Cells were subsequently washed in PBS, fixed and permeabilized using BD Cytofix/Cytoperm kit (BD Biosciences). Fixed islet cells were stained for insulin, glucagon and somatostatin at 4°C overnight using the following antibodies diluted in PermWash buffer (BD Biosciences): Alexa Fluor 488 mouse anti-somatostatin (BD Biosciences, 566032, 2 µg/100 µL), BV421 mouse anti-glucagon (BD Biosciences, 565891, 1 µg/100 µL) and APC rat anti-insulin (R&D Systems, C1417A, 2 µg/100 µL). After staining, islet cells were washed in PermWash buffer and resuspended in PBS supplemented with 5% FBS prior to flow cytometry. Flow cytometry was carried out on BD FACSCanto II (BD Biosciences). Samples were gated at 20 000–50 000 events. Compensation was performed post-acquisition using controls prepared from single-stained islet cell samples and TurboRFP-expressing islet cell samples. Data analysis and compensation were carried out using FCS Express 7 software (De Novo). Gating of endocrine cell populations was based on hormone marker staining. TurboRFP-positive population was gated based on FMO control.

### Glucose-stimulated insulin release

Prior to functional assay AAV8-transduced and non-transduced pseudoislets were kept for 1 h in a buffered solution (pH 7.4, containing 125 mM NaCl, 5.9 mM KCl, 2.6 mM CaCl_2_, 1.2 mM MgCl_2_, 25 mM HEPES, and 0.1% BSA) supplemented with 3 mM glucose. The experiments were carried out at 37°C in a humidified atmosphere with 5% CO_2_ using the same buffered solution as above. For assessment of basal and glucose-stimulated insulin secretion groups of 10–20 pseudoislets were first incubated for 1 h in the buffered solution containing 3 mM glucose and were subsequently moved to the buffered solution containing 15 mM glucose for 30 min. At the end of the experiment pseudoislets were lysed in M-PER protein extraction reagent (Thermo Fisher Scientific). Insulin concentration in assay supernatants and lysates was measured using AlphaLISA immunoassay (Perkin Elmer, AL3184). Amount of released insulin was recalculated to secreted per min and normalized to islet total insulin content.

### Image processing and analysis

Laser scanning confocal microscopy images were analyzed using Fiji software (Schindelin et al., 2012).

To compare TurboRFP expression in AAV8.CMV.TurboRFP-transduced islets and pseudoislets, fluorescence intensity was measured on the total intensity projection images of the z-stacks of each pseudo(islet). Native islets selected for this experiment were of similar size to the pseudoislets (170 ± 2 μm and 177 ± 7 µm in diameter, respectively). To estimate the extent of transduction in the islet core, TurboRFP-positive area was assessed in islet and pseudoislet optical sections taken at several layers below surface, using the same detection threshold for all images. To calculate TurboRFP-positive area as a fraction of the whole (pseudo)islet section area, TurboRFP-positive area was divided by the respective (pseudo)islet section area measured based on backscatter signal.

To measure AAV8 transduction efficiency, average fluorescence intensities per cell were calculated from the total intensity projection images of the control and AAV8.CMV.TurboRFP-transduced pseudoislet cells (total of 810 control and 933 AAV8-treated cells analyzed from 3 independent samples for mouse pseudoislets, and 889 control and 1112 AAV8-treated cells from 3 donors for human pseudoislets) dispersed into monolayers on chambered glass coverslips (Ibidi). Cells having average fluorescence intensity above that of the Mean +2 SDs of the control sample were considered positive for TurboRFP expression.

For quantification of the whole mount stainings we analyzed a minimum of 3 optical sections per pseudoislet separated by 15 µm distance, 6–9 pseudoislets from 2–3 independent experiments were analyzed per hormone stain. We analyzed a total of 1485 insulin-, 621 glucagon- and 319 somatostatin-labeled cells for the AAV8-transduced pseudoislets and 1387 insulin-, 368 glucagon- and 220 somatostatin-positive cells in the AdV5-transduced samples.

### Statistical analysis

Data processing and statistical analysis were performed using Excel (Microsoft) and Prism (Version 9, Graphpad). Data are presented as mean ± SEM unless otherwise indicated. Mann-Whitney test, two-way ANOVA or mixed-design ANOVA were used, as specified in figure legends. *p*-values <0.05 were considered statistically significant.

## Results

### Exposure to AAV8 during pseudoislet formation improves transduction efficiency

**FIGURE 1:**
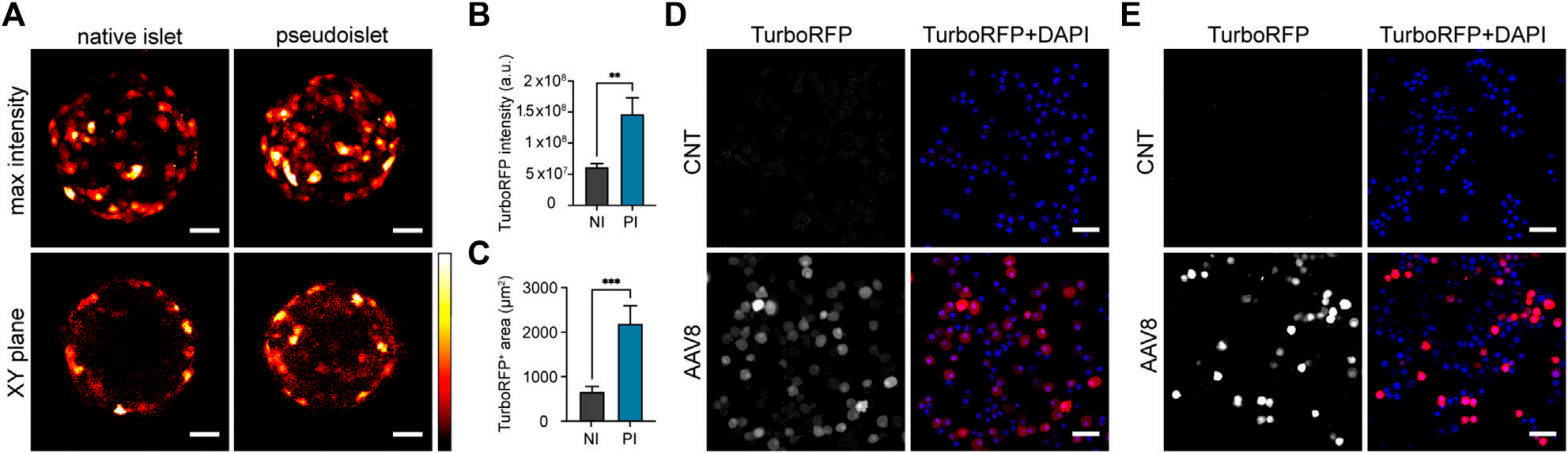
Exposure of islet cells to AAV8 during pseudoislet formation improves transduction efficiency. **(A)** Representative confocal images of native islets (NI) or pseudoislets (PI) transduced with AAV8.CMV.TurboRFP. Images were taken 2 weeks post-transduction. Max intensity = maximum intensity projection image of the whole pseudoislet or islet, XY plane = single plane image taken 50 µm from the islet surface. **(B)** TurboRFP intensity measured from total intensity projection images of AAV8-transduced islets and pseudoislets. a.u. = arbitrary units. **(C)** TurboRFP-positive area measured on the optical sections taken 50 µm deep from (pseudo)islet surface of AAV8-transduced islets and pseudoislets; **(D, E)** Representative confocal images of re-dispersed transduced (AAV8) and non-transduced (CNT) mouse **(D)** and human **(E)** pseudoislets. After pseudoislet formation cells were re-dispersed to assess transduction efficiency 2 weeks post-transduction. Images are single planes. Scale bars, 30 µm **(A)** and 40 µm **(D, E)**; *n* = 9–10, data presented as mean ± SEM, ** *p* < 0.01, *** *p* < 0.001, by Mann-Whitney U test **(B, C)**.

To assess whether AAV8 transduction efficiency can be improved during pseudoislet formation as compared to direct transduction of intact islets, we transduced native mouse islets and islet cell suspensions prepared with islets from the same isolation with 2.5 × 10^10^ viral genomes per ml (vg/mL) of AAV serotype 8 carrying TurboRFP under the CMV promoter (AAV8.CMV.TurboRFP). Native mouse islets were selected to be in the same size range as the expected final size of pseudoislets (170 ± 2 μm and 177 ± 7 µm in diameter, respectively). Based on total TurboRFP fluorescence intensity, transduction with AAV8 during pseudoislet formation resulted in a 2.5-fold higher expression as compared to that of intact isolated islets (Figures 1A, B). To assess AAV8-induced expression in the core region and throughout the (pseudo)islet, TurboRFP-positive areas were measured several cell layers below the surface. The fluorescent area was significantly larger in pseudoislets compared to that of native islets (Figures 1A, C, Supplementary Figure S1), indicating improved cell transduction across the organoid including the pseudoislet core.

AAV8 transduction efficiency in mouse and human pseudoislets was then assessed 2 weeks post-transduction by dispersing them into single cells and measuring average cell fluorescence intensity (Figures 1D, E). Using mean fluorescent intensity +2 SDs of cells from control non-transduced pseudoislets as a cut-off value, the transduction efficiency for AAV8.CMV.TurboRFP-transduced samples was estimated to be 77.5% ± 3.4% (*n* = 3) in mouse pseudoislets and 69.0% ± 11.6% in human pseudoislets (*n* = 3).

In order to check if transduction with AAV8 affects insulin secretory capacity of pseudoislets we have compared total insulin content, basal and glucose-stimulated insulin secretion in non-transduced and AAV8.CMV.TurboRFP-transduced islet organoids. We have found no effect of transduction with AAV8.CMV.TurboRFP on these functional parameters (Supplementary Figure S2).

### Kinetics of AAV8- and AdV5-driven TurboRFP expression in pseudoislets

To evaluate the kinetics of AAV8-driven expression in pseudoislets we transduced mouse islet cell suspensions with AAV8.CMV.TurboRFP and followed TurboRFP fluorescence intensity and mRNA levels in forming pseudoislets for 5 and 4 weeks post-transduction, respectively. We have used human adenovirus type 5 carrying TurboRFP under the CMV promoter (AdV5.CMV.TurboRFP) to compare AAV8-driven expression to that of AdV5. AdV5 concentration was chosen so that it provided comparable level of TurboRFP fluorescence to that from AAV8 10 days post-transduction. Although enzymatic dissociation of islets was temporarily accompanied with a certain percentage of dead cells, neither AdV5 nor AAV8, at chosen concentrations, caused any additional cell death in forming pseudoislets as compared to non-transduced samples (Supplementary Figure S3).

**FIGURE 2:**
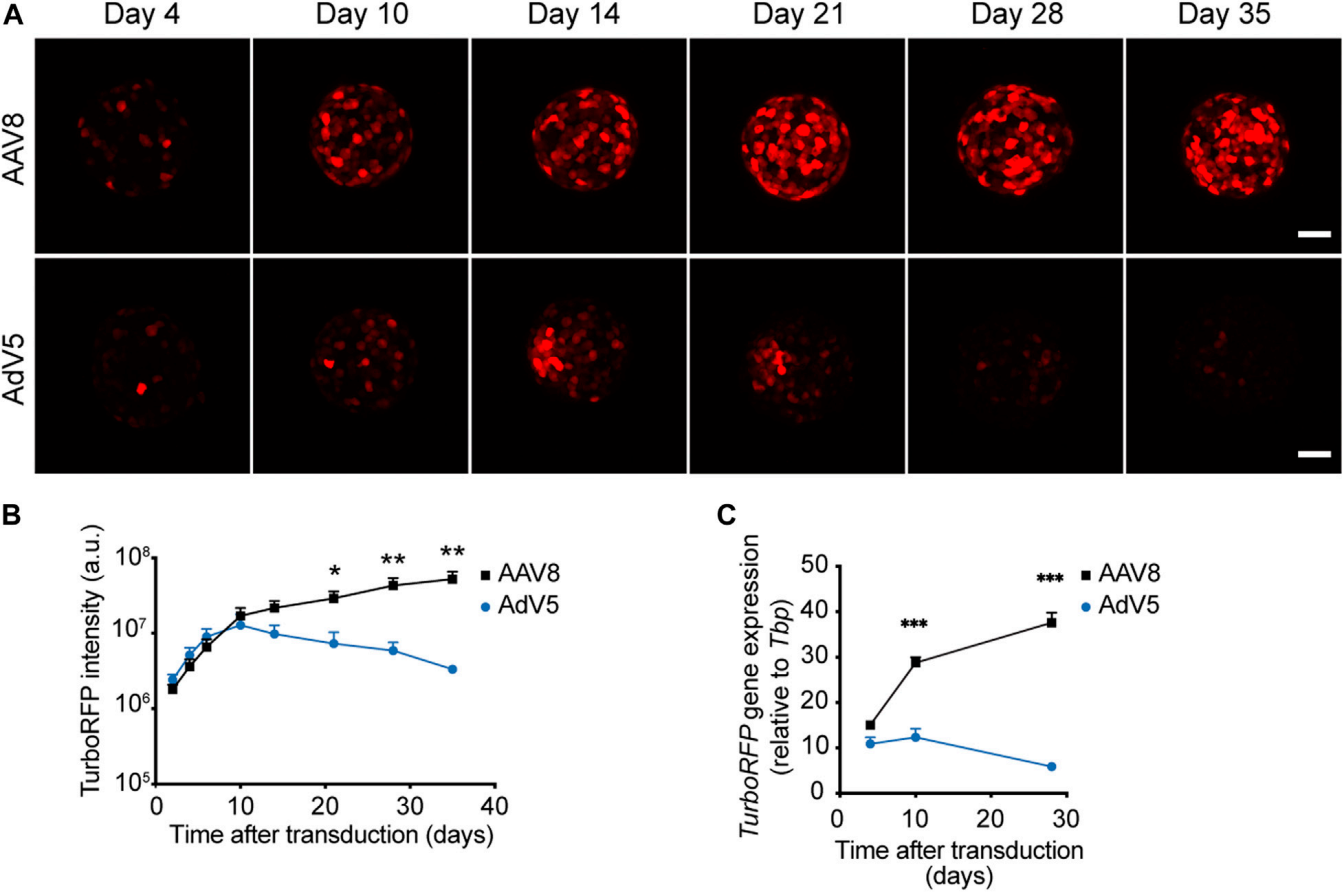
Kinetics of AAV8- and AdV5-induced expression in pseudoislets. **(A)** Representative confocal images of pseudoislets formed from AAV8.CMV.TurboRFP- or AdV5.CMV.TurboRFP-treated islet cell suspensions taken at different time points after transduction. **(B)** Kinetics of TurboRFP fluorescence intensity development in AAV8- and AdV5-transduced pseudoislets. TurboRFP intensity was calculated based on maximum intensity projection images. **(C)** Kinetics of TurboRFP mRNA levels in AAV8- and AdV5-transduced pseudoislets. All images are presented as maximum intensity projections. Scale bars, 50 μm; *n* = 9 **(B)** and 4 **(C)**; a.u. = arbitrary units. All data presented as mean ± SEM, * *p* < 0.01, ** *p* < 0.01, *** *p* < 0.001, by mixed-design ANOVA.

AAV8-induced TurboRFP expression continued to increase during the 5 weeks of the experiment, while the levels of AdV5-induced TurboRFP fluorescence intensity and mRNA reached their maximum approximately 2 weeks after transduction, and steadily declined afterwards (Figure 2).

### AAV8 and AdV5 tropism for endocrine cells within mouse pseudoislets

**FIGURE 3:**
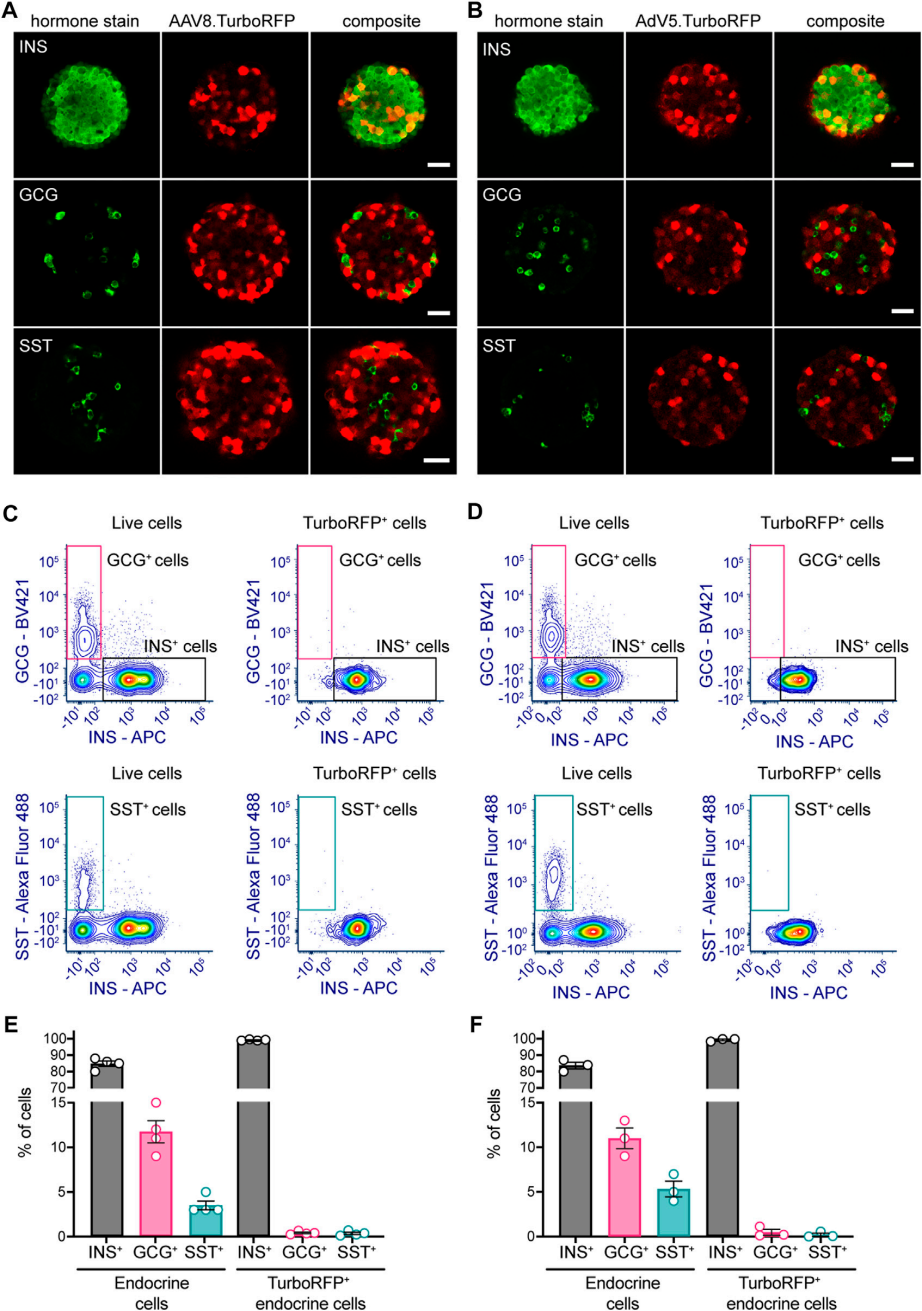
AAV8 and AdV5 endocrine cell tropism within mouse pseudoislets. Representative confocal images of **(A)** AAV8.CMV.TurboRFP- or **(B)** AdV5.CMV.TurboRFP-transduced pseudoislets. Pseudoislets were immunostained for insulin (INS), glucagon (GCG) or somatostatin (SST) to evaluate TurboRFP localization in major endocrine cell types. Representative flow cytometric data for AAV8.CMV.TurboRFP- **(C)** and AdV5.CMV.TurboRFP-transduced **(D)** pseudoislets. Panels on the left of **(C, D)** demonstrate insulin-, glucagon- (upper left) and somatostatin-positive cell (lower left) distribution within the entire live cell population, and respective panels on the right of **(C, D)** show distribution of the same within the TurboRFP-positive population. Data from several independent pseudoislet preparations are summarized in panel **(E)** (AAV8.CMV.TurboRFP-transduced pseudoislets) and panel **(F)** (AdV5.CMV.TurboRFP-transduced pseudoislets). Confocal images are single planes. Scale bars, 30 µm. 6-9 pseudoislets were processed for each hormone immunostaining **(A, B)**. Data presented as mean ± SEM, *n* = 3–4 **(E, F)**.

To study AAV8 and AdV5 cell tropism towards the main endocrine cells present within the islet cell suspension during pseudoislet formation, we have examined TurboRFP-positive cells in transduced pseudoislets immunostained for insulin (β-cells), glucagon (α-cells) or somatostatin (δ-cells) (Figures 3A, B). Pseudoislets were stained for one hormone at a time to optimize further image acquisition and analysis. For quantification of stained and transduced cells we analyzed a minimum of 3 optical sections per pseudoislet separated by 15 µm distance, 6–9 pseudoislets were analyzed per hormone stain. In AAV8-transduced pseudoislets 99.6% ± 0.2% of TurboRFP-expressing cells were positive for insulin, while only 0.8% ± 0.3% and 0.3% ± 0.1% of TurboRFP-positive cells were labeled as glucagon- and somatostatin-positive in samples stained for these hormones. This was very similar to the distribution observed in AdV5.CMV.TurboRFP-transduced pseudoislets where 99.3% ± 0.3% of TurboRFP-positive cells were insulin-positive and less than 0.5% of TurboRFP-positive cells co-labeled for glucagon or somatostatin (0.1% ± 0.1% and 0.2% ± 0.2%, respectively).

Given the very small number of somatostatin and glucagon cells positive for TurboRFP, we sought to confirm our IHC and confocal imaging data by flow cytometry analysis.

Flow cytometry data corroborated IHC results showing that both viral vectors primarily induced TurboRFP expression in insulin-producing β-cells (Figures 3C–F). In both AAV8.CMV.TurboRFP- and AdV5.CMV.TurboRFP-transduced pseudoislets over 99% of endocrine TurboRFP-positive cells were positive for insulin, with glucagon- and somatostatin-positive cells each contributing less than 0.5% to the TurboRFP-positive population. Considering that glucagon and somatostatin cells represented respectively ∼10% and ∼5% of the entire pseudoislet endocrine cell population (Figures 3E, F), these cells were clearly underrepresented in the TurboRFP-positive population.

### Simultaneous transduction with AdV5 substantially enhances AAV8-induced expression in pseudoislets

**FIGURE 4:**
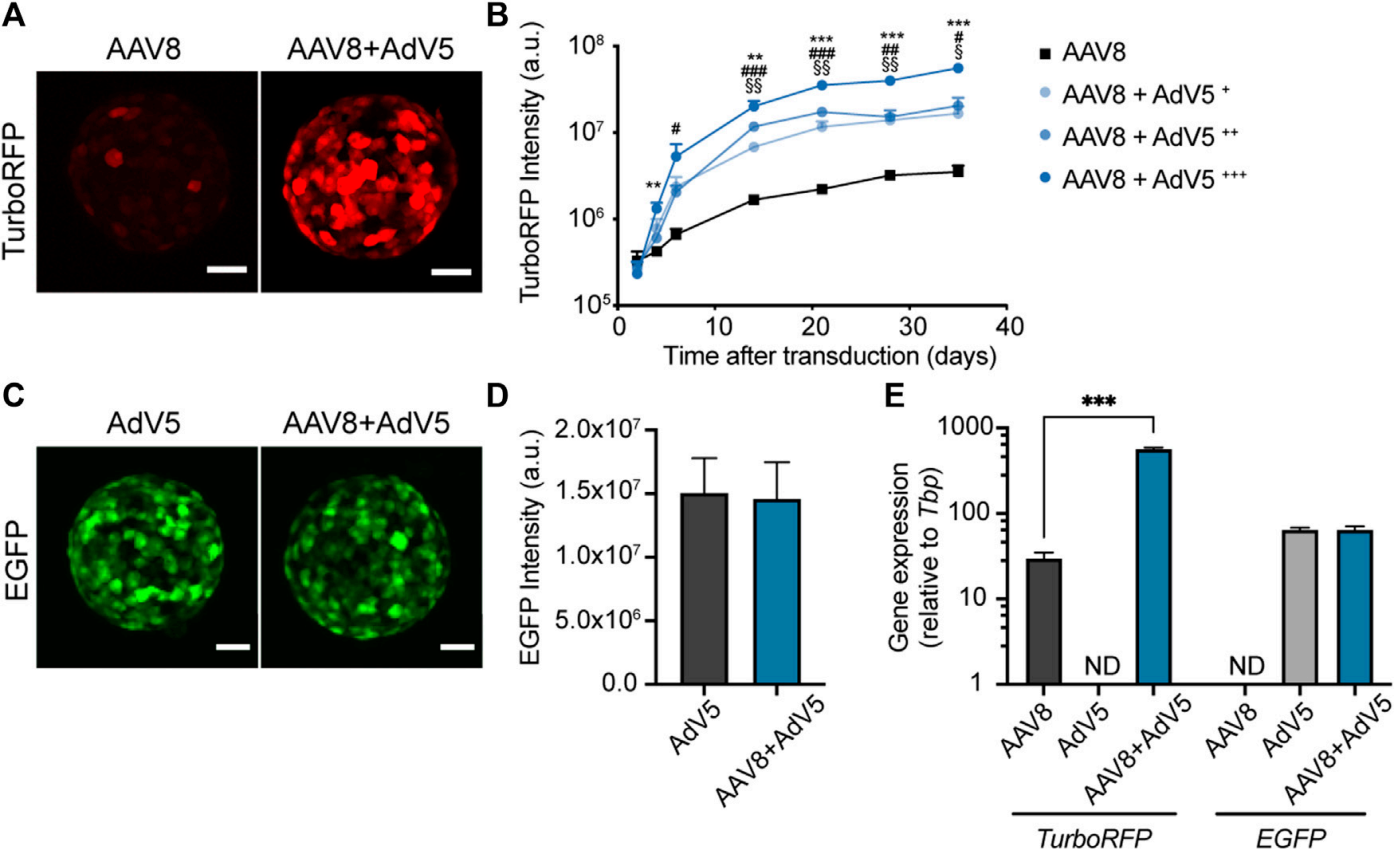
Simultaneous transduction with AdV5 substantially enhances AAV8-induced expression in pseudoislets. **(A)** Representative confocal images showing enhanced TurboRFP expression in pseudoislets formed from islet cell suspensions transduced with a combination of AAV8.CMV.TurboRFP and AdV5.CMV.EGFP (AAV8+AdV5) compared to AAV8.CMV.TurboRFP alone (AAV8). **(B)** Kinetics of TurboRFP fluorescence intensity development in pseudoislets transduced with AAV8.CMV.TurboRFP alone or in combination with varying amounts of AdV5.CMV.EGFP (AAV8+AdV5+ = 8.5 × 104 PFU/mL, AAV8+AdV5++ = 1.7 × 105 PFU/mL and AAV8+AdV5+++ = 8.5 × 105 PFU/mL). TurboRFP intensity was calculated based on maximum intensity projection images. **(C, D)** Simultaneous transduction with AAV8.CMV.TurboRFP does not affect EGFP fluorescence expression induced by AdV5.CMV.EGFP as illustrated by representative confocal images **(C)** and quantification of EGFP fluorescence intensity **(D)**. EGFP fluorescence intensity was calculated based on maximum intensity projection images taken on day 10–14 after transduction. **(E)** TurboRFP and EGFP mRNA levels measured 2 weeks post-transduction confirm increased expression of AAV8-induced TurboRFP in pseudoislets co-transduced with AdV5, while AdV5-induced EGFP gene expression was not affected by the presence of AAV8.CMV.TurboRFP. All images are maximum intensity projections, scale bars, 40 µm **(A, C)**. All data presented as mean ± SEM; *n* = 6 **(B)**, 9 **(D)** and 3 **(E)**. ** *p* < 0.01, *** *p* < 0.001 between groups AAV8 and AAV8+AdV5^+++^, ^#^ *p* < 0.05, ^##^ *p* < 0.01, ^###^ *p* < 0.001 between groups AAV8 and AAV8+AdV5^++^, § *p* < 0.05, §§ *p* < 0.01 between groups AAV8 and AAV8+AdV5^+^, by mixed-design ANOVA **(B)**, by Mann-Whitney U test **(D)**, *** *p* < 0.001, by two-way ANOVA **(E)**.

Previous studies on intact islets have shown that AAV transduction can be improved by simultaneous transduction with AdV (Craig et al., 2009). However, optimal AdV concentrations and the effect of co-transduction on expression kinetics in islet cells are not known. To study the effect of co-transduction with AdV on the levels and kinetics of AAV8-driven expression in pseudoislets we have transduced islet cell suspensions with AAV8.CMV.TurboRFP alone or in combination with varying amounts (8.5 × 10^4^ PFU/mL, 1.7 × 10^5^ PFU/mL and 8.5 × 10^5^ PFU/mL) of human adenovirus type 5 carrying EGFP under the CMV promoter (AdV5.CMV.EGFP), and followed TurboRFP fluorescence intensity in forming pseudoislets for 5 weeks post-transduction (Figure 4).

Co-transduction with AdV5 substantially amplified AAV8-driven TurboRFP expression without changing the kinetics of TurboRFP fluorescence intensity development (Figures 4A, B). Increase in AAV8-driven TurboRFP expression correlated with the amount of AdV5 used for co-transduction. The presence of AAV8.CMV.TurboRFP did not have any effect on AdV5-induced EGFP fluorescence intensity (Figures 4C, D). At the gene expression level, we observed that enhancement of TurboRFP fluorescence intensity was in line with the 20-fold increase in *TurboRFP* mRNA amount in pseudoislets transduced with a mixture of AAV8.CMV.TurboRFP and AdV5.CMV.EGFP as compared to AAV8.CMV.TurboRFP alone. Also, we could confirm that *EGFP* expression was not affected by co-transduction with AAV8.CMV.TurboREF (Figure 4E).

Transduction of human islet cell suspensions with AAV8.CMV.TurboRFP alone or in combination with 8.5 × 10^5^ PFU/mL of AdV5.CMV.EGFP demonstrated that AAV8-driven expression in human pseudoislets follows similar kinetics to that observed in mouse pseudoislets and could also be significantly enhanced by co-transduction with AdV5, with TurboRFP fluorescence intensity levels in a similar range to that seen in mouse pseudoislets (Supplementary Figure S4).

## Discussion

In this study we provide detailed characterization of pseudoislet transduction mediated by AAV8. We describe AAV8 efficiency, tropism towards major islet endocrine cell types and kinetics of AAV8-introduced gene expression and compare most of these parameters with those of AdV5.

Our results demonstrate that transduction with AAV8 during pseudoislet formation allows to achieve substantially higher expression levels of the introduced gene compared to native islets. Previously we have demonstrated similar improvement in AdV transduction efficiency when AdV5 was used to transduce reaggregating pseudoislets (van Krieken et al., 2019). Even though the relatively small size of AAV viral particles compared to AdV (∼25 nm vs. ∼100 nm) (Kennedy and Parks, 2009; Horowitz et al., 2013) may allow better penetration into the core of native islets, the difference in transduction levels within the central area between intact islets and pseudoislets was substantial. Thus, pseudoislets offer the possibility to achieve a uniform gene transfer with AAV throughout the organoid. This is in contrast to disproportionate transduction of the outer cell layers following native islet transduction. Successful gene transfer within the inner regions of the (pseudo)islet is especially valuable for further transplantation. Cells laying within the islet core are the ones that suffer the most from shortage of oxygen and nutrient supply before full revascularization takes place (Komatsu et al., 2017), and consequently are the ones to potentially benefit the most from being equipped with protective elements via genetic modification.

Exposure of islet cells to the virus while in suspension allows the virus to interact with all the cells within the forming pseudoislet to the same extent whereas in intact islets the outer cell layers are the ones exposed the most. In native mouse islets, glucagon-producing cells are situated in the mantle layer of the islet while insulin-secreting cells lay within the core region (Cabrera et al., 2006; Steiner et al., 2010). The glucagon-producing cells located in the islet periphery in intact islets are therefore exposed to higher number of viral particles per cell than when cell suspension is used. Consequently, dispersing islet cells prior to transduction affects not only efficiency of transduction but also the probability of different islet cell types to encounter the virus. When transducing re-aggregating islet cells, we found that both AdV5 and AAV8 mostly transduced β-cells with very few α- and δ-cells expressing virus-introduced TurboRFP. Our results are in line with previous works that have evaluated AAV tropism towards α- and β-cells in native islets (Craig et al., 2009; Jimenez et al., 2011). Thus, α-cells appear to be less readily transduced by AAVs irrespective of the mode of exposure to the virus. Although what may be causing lower efficiency of AAV in glucagon-producing cells has not been determined, the low level of transduction of α-cells by adenoviral vectors was proposed to be related to the ability of glucagon cells to rapidly eradicate the virus resulting in much lower levels of translation of the cargo gene compared to β-cells (Marroqui et al., 2015). It is well established that β-cells are particularly susceptible to various viral infections including transduction with adenoviral vectors (Ifie et al., 2018). Inferior protection of insulin-producing cells against viruses is even regarded as one of potential triggers for the development of the β-cell targeted autoimmune response and subsequent type 1 diabetes (Op de Beeck and Eizirik, 2016).

To the best of our knowledge, transduction of somatostatin-producing cells was not previously evaluated and our study is the first to look at AdV and AAV tropism towards δ-cells. Somatostatin cells constitute a relatively sparse islet cell population, accounting for about 3%–5% of islet cells in rodents (Steiner et al., 2010). The low number of somatostatin-positive cells within the islet may present a problem for proper evaluation of transduction within this cell population. To reliably assess the cell tropism of viral vectors we have used two alternative approaches, IHC and flow cytometry, with both yielding very similar results. We have consistently detected a small number of transduced δ-cells both when AdV5 and AAV8 vectors were used, indicating that in principle somatostatin cells can also be infected by these viruses. However, even accounting for the small percentage of δ-cells within islet cell suspension, AdV5 and AAV8 have disproportionally greater ability to transduce β-cells.

The activity of a promoter in a given cell type defines the levels and visibility of a vector-introduced gene expression. To ensure transgene expression in all islet cells we have used ubiquitous CMV promoter. The CMV promoter conveys strong expression in islets in general (Londrigan et al., 2007), but its activity in individual islet cell types has not been studied. Hence, we cannot fully exclude that cell-specific differences in CMV promoter activity contributed to the visible transduction differences in islet cell types.

AAV transduction efficacy towards the same cell type often varies from species to species (Loiler et al., 2003; Asokan et al., 2012; Lisowski et al., 2014) and can thus differ between human and mouse islet cells. Due to low availability of human material we could only conduct a limited number of experiments with human pseudoislets. Nevertheless, the kinetics experiments demonstrate that AAV8-induced TurboRFP in human islet organoids follows extended expression kinetics similar to that seen in mouse pseudoislets, reaching equal levels of TurboRFP fluorescence with the possibility to enhance expression using AdV. The transduction efficiency of AAV8 in human pseudoislets was comparable to that detected in mouse pseudoislets. Thus, the pseudoislet transduction methodology described here allows to achieve a relatively high efficiency of AAV8-mediated gene transfer into human islet cells, in a similar range to that seen in mouse islet organoids.

Our previous work shows that AdV-mediated transduction is not well maintained after *in vivo* transplantation (van Krieken et al., 2019), while it was shown by others that AAV-induced expression in pancreatic islets is preserved *in vivo* for at least 6 months (Wang et al., 2006; Jimenez et al., 2011). Our current results demonstrate that under *in vitro* culture, pseudoislets maintain a high level of AAV8-induced TurboRFP expression throughout the whole 5 weeks of the experiment. This is in contrast with AdV5 that only induced relatively short-lived expression with a maximum at ∼10 days post-transduction that substantially faded by 4 weeks. Thus, AAVs have an evident advantage over AdVs in *ex vivo* pseudoislet transduction if long-term expression is desired.

Efficiency of transduction with AAV can be improved by co-infection with a helper virus such as AdV (Fisher et al., 1996; Samulski and Muzyczka, 2014). In line with previous research on other cell types (Fisher et al., 1996), our data show enhancement of AAV-driven expression in islet cells proportional to the amount of the AdV used. Presence of AAV did not affect AdV-driven expression indicating that these two viruses can be used together in pseudoislets. This is true not only for enhancement of AAV-induced gene expression but also for simultaneous introduction of several genes when short-term expression provided by AdV is desired.

In conclusion, we have described and characterized a method for *in vitro* pseudoislet transduction with AAV8 that allows achieving efficient and stable expression of a gene of interest in insulin-producing cells. Detailed data provided in our work can serve as a reference point for preclinical studies looking at the effects of islet modifications on islet function or transplantation outcomes. Several recent studies have looked at ways to modify AAV capsids in order to increase efficiency of transduction in pancreatic islets (Chen et al., 2017; Pekrun et al., 2019). Noteworthy is, that there are yet other means to improve virus-induced expression such as, for example, promoter and transgene optimization (Li and Samulski, 2020). The procedure described in our work could be used alongside other methods aiming at optimizing transduction efficacy, with the objective of manufacturing improved tissue for transplantation. Enhanced AAV transduction efficiency combined with other advantages of pseudoislets, such as better survival and functionality (Yu et al., 2018), makes *ex vivo* modified pseudoislets a unique and promising tool for clinical transplantation.

## Data Availability

The raw data supporting the conclusion of this article will be made available by the authors, without undue reservation.
