# Supplementary material for: Sustained heterologous gene expression in pancreatic islet organoids using adeno-associated virus serotype 8
Source: Front Bioeng Biotechnol. 2023 Jul 19;11:1147244. doi: 10.3389/fbioe.2023.1147244 (PMC10400289; doi:10.3389/fbioe.2023.1147244)
Supplement: Supplementary file 1 [file DataSheet1.pdf]

Supplemental information

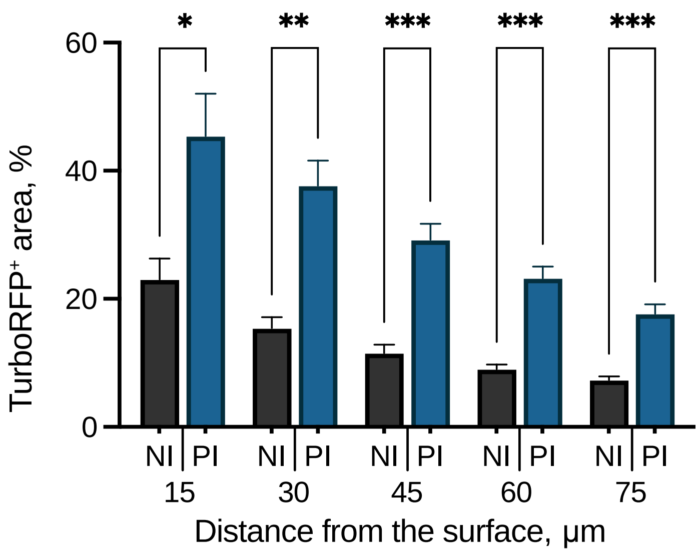

Supplementary figure 1. Relative TurboRFP-positive area in native islets and pseudoislets transduced with AAV8.CMV.TurboRFP

TurboRFP-positive area was measured on the optical sections taken 15-75 µm deep from the surface of AAV8-transduced native islets (NI) and pseudoislets (PI) using the same detection threshold, and divided by the whole pseudo(islet) section area. Note that TurboRFP-positive area measurements are limited by excitation light penetration and weaker detection of emitted fluorescence from the deeper layers of the (pseudo)islet. Average fold difference between NI and PI TurboRFP-positive area, % is 2.69 ± 0.02. Data presented as mean ± SEM; n=9-10, * $p< 0.05$, ** $p< 0.01$, *** $p< 0.001$, by mixed-design ANOVA.

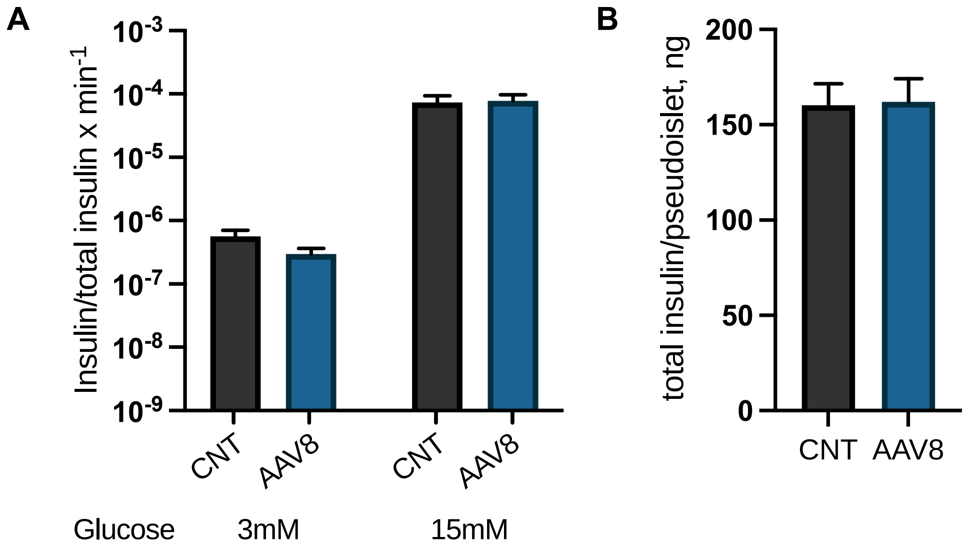

Supplementary figure 2. Transduction with AAV8.CMV.TurboRFP does not affect glucose-stimulated insulin release and total insulin content of pseudoislets.
Groups of non-transduced pseudoislets (CNT) and pseudoislets transduced with AAV8.CMV.TurboRFP (AAV8) were incubated under static conditions in buffered solutions containing 3mM and 15mM glucose (A). At the end of the incubation pseudoislets were lysed to measure total insulin content (B). No significant differences by two-way ANOVA (A) or Mann-Whitney U test (B). Data presented as mean ± SEM; n=7 (A, B).

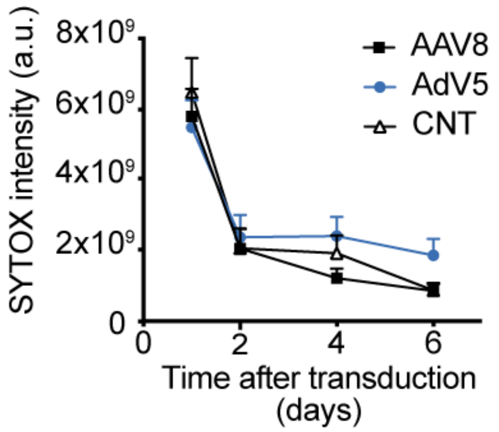

Supplementary figure 3. Transduction with AAV8.CMV.TurboRFP and AdV5.CMV.TurboRFP does not affect islet cell survival.

Dead islet cells were labeled with SYTOX green. SYTOX green fluorescence intensity was calculated based on total intensity projection images. No significant differences by mixed design ANOVA. Data presented as mean ± SEM; n=9.

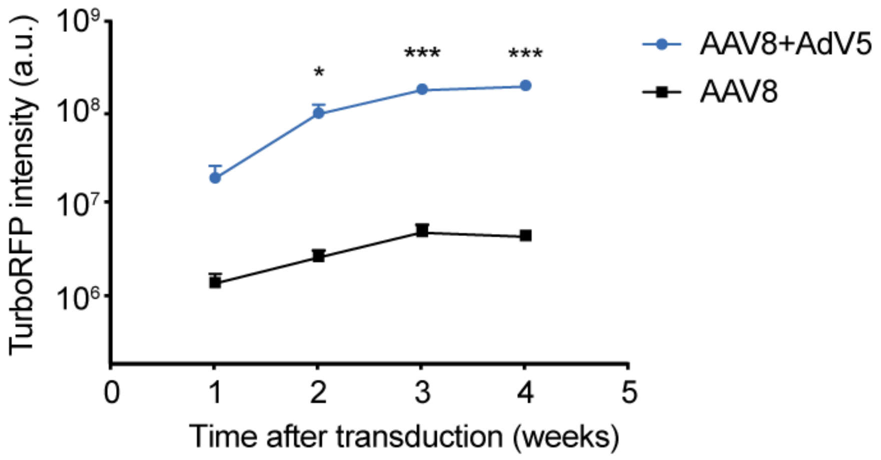

Supplementary figure 4. Kinetics of AAV8-induced expression in human pseudoislets and its enhancement by AdV5.

Kinetics of TurboRFP fluorescence intensity development in human pseudoislets transduced with AAV8.CMV.TurboRFP alone (AAV8) or in combination with 8.5×10^5 PFU/ml of AdV5.CMV.EGFP (AAV8+AdV5). TurboRFP intensity was calculated based on maximum intensity projection images. Data presented as mean ± SEM; n=6, * $p < 0.05$, *** $p < 0.001$, by mixed-design ANOVA.
